# Depressive and anxiety disorders worsen the prognosis of glioblastoma

**DOI:** 10.18632/aging.103593

**Published:** 2020-10-28

**Authors:** Xiaojun Fu, Chenxing Wu, Ning Han, Ning Liu, Song Han, Xuebin Liu, Shouwei Li, Changxiang Yan

**Affiliations:** 1 Sanbo Brain Hospital, Capital Medical University, Beijing, PR China; 2 Capital Medical University, Beijing, PR China; 3 Department of Neurosurgery, Chinese PLA Tianjin Rehabilitation and Recuperation Center of Joint Service Support Force, Tianjin, PR China; 4 Zhong Guang Tianyi Bio Technology Co., Ltd., Beijing, China

**Keywords:** glioblastoma, depression, major depressive disorder, anxiety, patient outcomes

## Abstract

Glioblastoma multiforme (GBM) is one of the most malignant tumors. Depressive and anxiety disorders may co-exist with GBM. We investigated whether depression and anxiety influenced the outcomes of GBM. The Patient Health Questionnaire 9-item (PHQ-9) and Generalized Anxiety Disorder 7-item (GAD-7) scales were used to investigate the mental condition of GBM patients in our department, and the overall survival times of these patients were monitored. The scores on both scales were higher in GBM patients than in healthy controls. For each scale, GBM patients were divided into high- and low-score groups based on the average score. The prognosis was poorer for GBM patients in the high-score groups than for those in the low-score groups. Moreover, magnetic resonance imaging revealed that tumor necrosis was more prevalent among high-scored GBM patients. Cellular experiments were performed on primary GBM cells from patients with either high or low scores on both scales. Sphere formation, EdU and wound healing assays revealed greater proliferation and invasion capacities in GBM cells from patients with high scores on both scales. Western blotting assay revealed significantly different expression of epithelial and mesenchymal markers between the two groups. Thus, our analysis revealed a clinically important correlation between depression/anxiety and GBM prognosis.

## INTRODUCTION

Glioblastoma multiforme (GBM) is the most common primary World Health Organization grade IV brain tumor type in adults [1]. Tens of thousands of GBM cases are confirmed every year [2]. GBM is also the most malignant type of tumor in the central nervous system, with a median survival time of only 18-24 months. Approximately 13,000 patients in the US die of GBM each year [2, 3]. GBM patients have to endure multiple forms of physical and psychological stress and pain. Heterogeneity is an important feature of GBM, which may contribute to differences in severity, chemo/radio-sensitivity, prognosis and even behavioral and mental health among GBM patients [1, 4].

As two of the most common psychological disorders, depression and anxiety have caused morbidity and mortality in millions of people worldwide [5]. Many biological [6], psychological [7] and social environmental factors [8] are involved in the pathogenesis of depression and anxiety. Psychological factors can also impact an individual’s physical health in a variety of ways. For example, depressive and anxiety disorders have been reported to co-exist with cardiovascular disease [9], traumatic injuries [10] and many cancer types [11–13].

It seems rather intuitive that cancer patients would be negatively impacted by their diagnoses. Chronic pain caused by both the cancer and its treatment can also result in emotional changes or even mental disorders [14–16]. On the other hand, mental disorders (especially depressive and anxiety disorders) can alter the production of inflammatory molecules, cytokines and chemokines, the metabolism of neurotransmitters, the function of the neuroendocrine system, etc. [9, 13, 17]. These factors are also recognized as possible contributors to the development and heterogeneity of GBM [3, 18]. Depression and anxiety seem to co-exist with GBM more often than with other cancers [12]. However, until now, the possible relationship between depression/anxiety and GBM has not been explored.

Here, we analyzed the relationship between GBM prognosis and depression/anxiety in 84 GBM patients. We surveyed these patients through two commonly used questionnaires: the Patient Health Questionnaire 9-item (PHQ-9) and Generalized Anxiety Disorder 7-item (GAD-7) scales. We then analyzed the relationship between PHQ-9/GAD-7 scores and overall survival times in both male and female patients. To explore the reasons for the prognostic differences among these patients, we performed magnetic resonance imaging (MRI) and tested the proliferative and invasive capacities of GBM cells from the different groups *in vitro*. Our findings have helped to clarify the complex relationship between GBM and mental disorders.

## RESULTS

### Questionnaires reveal significant depressive and anxiety disorders in GBM patients

**Figure 1:**
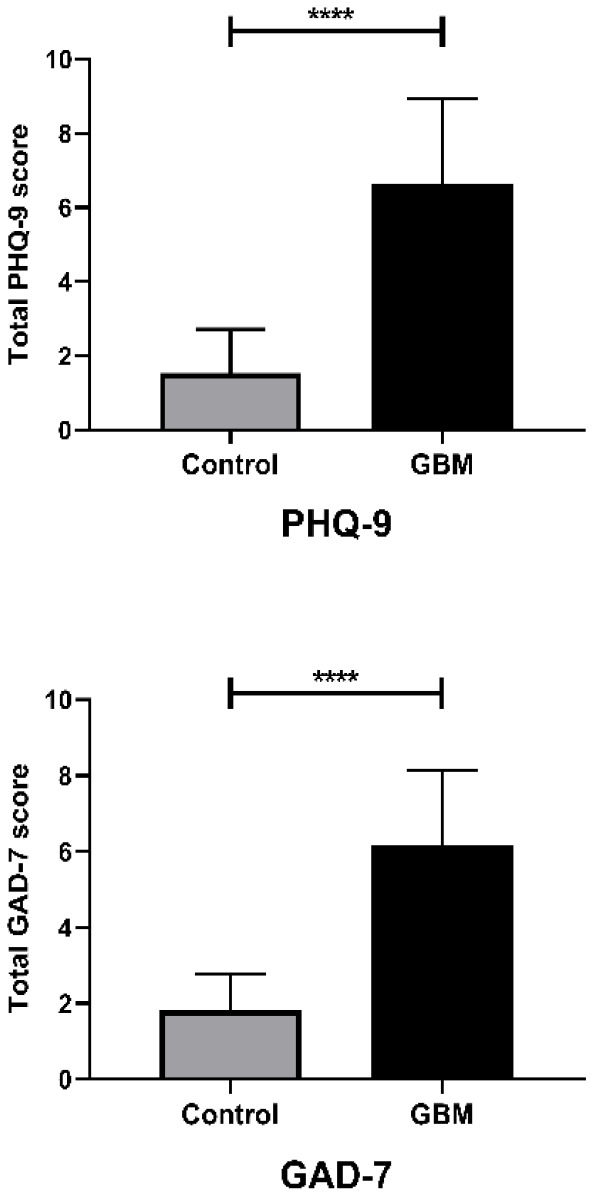
**Questionnaire results differed between GBM patients and controls, and between genders.** Bar plot comparing the PHQ-9 and GAD-7 questionnaire results between the control and GBM groups; ****, *P* < 0.0001. Student’s t-test was used for statistical analysis.

First, we used the PHQ-9 and GAD-7 scales (Tables 1 and 2) to assess depression and anxiety in our enrolled patients and a group of healthy controls. The basic information of all enrolled patients was provided as Supplementary Table 1. As shown in Table 3 and Figure 1, after learning about their diagnoses, the GBM patients all exhibited significantly greater depression and anxiety than their control conterparts. We then examined the severity distribution of the PHQ-9 and GAD-7 scores in GBM patients. As shown in Table 4, 90% of GBM patients displayed minimal to mild depressive disorder, and 97.6% displayed minimal to mild anxiety disorder. Eight (10%) and two (2.4%) patients exhibited moderate depression and anxiety, respectively. The more detailed PHQ-9 and GAD-7 scores are provided in Supplementary Table 2.

**Table 1:** Details of the patient health questionnaire 9-item (PHQ-9) scale.

**No.**	**Items**
**1.**	Little interest or pleasure in doing things
**2.**	Feeling down, depressed, or hopeless
**3.**	Trouble falling or staying asleep, or sleeping too much
**4.**	Feeling tired or having little energy
**5.**	Poor appetite or overeating
**6.**	Feeling bad about yourself or that you are a failure or have let yourself or your family down
**7.**	Trouble concentrating on things, such as reading the newspaper or watching television
**8.**	Moving or speaking so slowly that other people could have noticed. Or the opposite: being so fidgety or restless that you have been moving around a lot more than usual
**9.**	Thoughts that you would be better off dead, or of hurting yourself

The PHQ-9 scale is provided as a 9-item questionnaire. Each item is scored on four levels (0, 1, 2 and 3). The total PHQ-9 score (the sum of all items) indicates the depression severity: I: 1-4 Minimal depression; II: 5-9 Mild depression; III: 10-14 Moderate depression; IV: 15-19 Moderately severe depression; V: 20-27 Severe depression.

**Table 2:** Details of the generalized anxiety disorder 7-item (GAD-7) scale.

**No.**	**Items**
**1.**	Feeling nervous, anxious, or on edge
**2.**	Not being able to stop or control worrying
**3.**	Worrying too much about different things
**4.**	Trouble relaxing
**5.**	Being so restless that it’s hard to sit still
**6.**	Becoming easily annoyed or irritable
**7.**	Feeling afraid as if something awful might happen

The GAD-7 scale is provided as a 7-item questionnaire. Each item is scored on four levels (0, 1, 2 and 3). The total GAD-7 score (the sum of all items) indicates the severity of anxiety: I: 1-4 Minimal Anxiety; II: 5-9 Mild Anxiety; III: 10-14 Moderate Anxiety; IV: 15-21 Severe Anxiety.

**Table 3:** Comparison of PHQ-9 and GAD-7 scores between diagnosed GBM patients and control subjects.

	**GBM patients (n = 84)**	**Controls (n = 45)**	**95% CI**	***P* value**
**PHQ-9**	6.62 ± 0.25	1.53 ± 0.18	4.357 to 5.815	<0.0001
**GAD-7**	6.17 ± 0.21	1.82 ± 0.14	3.730 to 4.958	<0.0001

Data are presented as the mean ± standard error of the mean. The differences between the two groups were analyzed with unpaired sample t-tests. CI: confidence interval between the GBM and Control groups.

**Table 4:** The severity distribution of PHQ-9 and GAD-7 scores in GBM patients.

**Scale**	**Severity**	**Number of Cases**	**Percentage**
**PHQ-9**	I	16	19%
	II	60	71%
	III	8	10%
	IV	0	0
	V	0	0
**GAD-7**	I	16	19%
	II	66	79%
	III	2	2%
	IV	0	0

Severity levels I to V correspond to different ranges of PHQ-9 and GAD-7 questionnaire scores (see Tables 1 and 2).

### The relationship between the questionnaire results and prognosis in GBM patients

**Figure 2:**
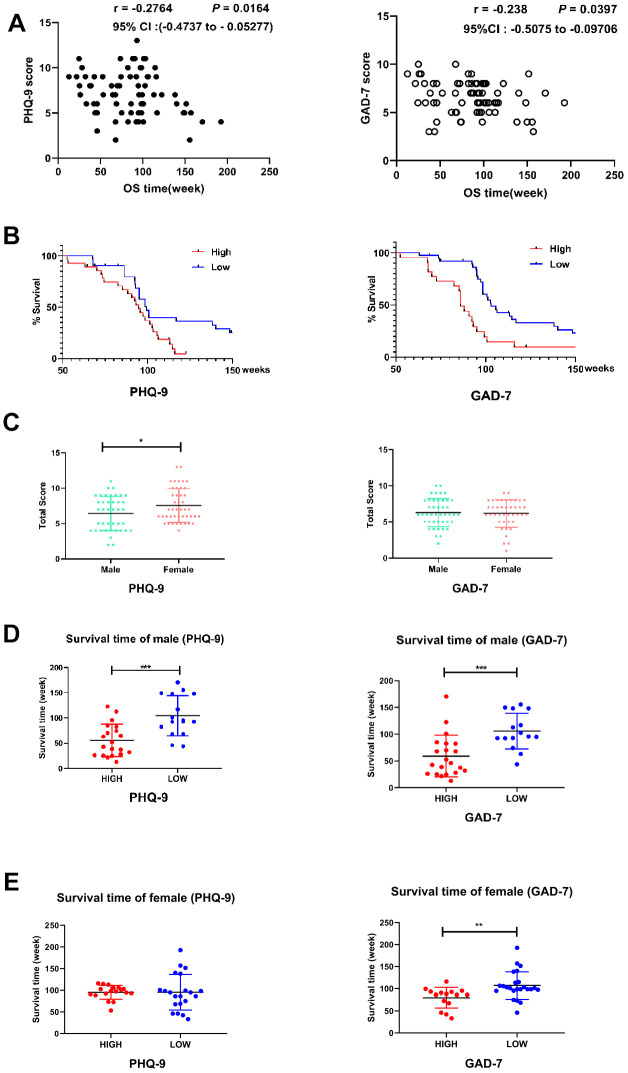
**Correlation between the questionnaire results and GBM outcomes.** (**A**) Scatter plots demonstrating the correlations of PHQ-9 (left) and GAD-7 (right) scores with the overall survival (OS) times in GBM patients. Pearson correlation analysis was used. The correlation coefficient, 95% confidence interval and *P* value are provided in the upper right corner of each plot. (**B**) Kaplan-Meier analyses of OS in patients with high or low PHQ-9 (left side) or GAD-7 (right side) scores. The OS times differed significantly between patients with higher (red line, n = 38) and lower (blue line, n = 36) PHQ-9 scores; *P* = 0.0172. The OS times also differed significantly between patients with higher (red line, n = 36) and lower (blue line, n = 39) GAD-7 scores; *P* = 0.002. A log-rank (Mantel-Cox) test was used. (**C**) Histogram displaying the differences in PHQ-9 (left side, n ^male^ = 40, n ^female^ = 44, *P* = 0.0378) and GAD-7 (right side, n ^male^ = 40, n ^female^ = 44, *P* = 0.5742) results between male and female GBM patients. Student’s t-test was used for statistical analysis. *, *P* < 0.05. (**D**) Histogram displaying the differences in OS between male GBM patients with high or low PHQ-9 or GAD-7 scores. The OS times differed significantly between male patients with high (red, n = 20) and low (blue, n = 16) PHQ-9 scores (left side); *P* = 0.0003. The OS times also differed significantly between male patients with high (red, n = 21) and low (blue, n = 15) GAD-7 scores (right side); *P* = 0.0006. Student’s t-test was used for statistical analysis; ***, *P* < 0.001. (**E**) Histogram displaying the differences in OS between female GBM patients with high or low PHQ-9 or GAD-7 scores. The OS times did not differ significantly between female patients with high (red, n = 18) and low (blue, n = 21) PHQ-9 scores (left side); *P* = 0.9966. The OS times differed significantly between female patients with high (red, n = 15) and low (blue, n = 24) GAD-7 scores (right side); *P* = 0.0054. Student's t-test was used for statistical analysis; **, *P* < 0.01.

To evaluate the relationship between the PHQ-9/GAD-7 scores and prognoses of our GBM patients, we determined their overall survival times using telephone calls or chat tools. All the patients had undergone brain surgery with the same group of doctors. As shown in Figure 2A, the PHQ-9 and GAD-7 scores both exhibited significant negative correlations with the overall survival time.

To further examine this relationship, we divided GBM patients into “high” and “low” groups according to whether their PHQ-9 scores were higher or lower than the average. Likewise, we divided the patients into “high” and “low” groups according to whether their GAD-7 scores were higher or lower than the average. In total, 22 patients had higher-than-average scores on both the PHQ-9 and GAD-7 scales (Supplementary Figure 1), while 27 patients had lower-than-average scores on both scales (Supplementary Figure 2). Cox regression analyses were used to evaluate the mortality of patients in the high and low groups for each scale. As shown in Figure 2B, GBM patients with higher PHQ-9 or GAD-7 scores exhibited poorer survival than their lower-score counterparts.

It is well established that men and women react significantly differently to similar psychological factors. As shown in Figure 2C, male patients had significantly lower PHQ-9 scores than female patients, while there was no significant difference in GAD-7 scores between the genders. The effects of depression/anxiety on the overall survival time differed between the genders. Male patients with higher PHQ-9 and GAD-7 scores exhibited shorter overall survival times than male patients with lower scores (Figure 2D). However, female patients with higher PHQ-9 scores did not exhibit significantly shorter overall survival times than female patients with lower scores, while female patients with higher GAD-7 scores did display shorter overall survival times than female patients with lower scores (Figure 2E).

### MRI patterns of the high and low PHQ-9 and GAD-7 groups

**Figure 3:**
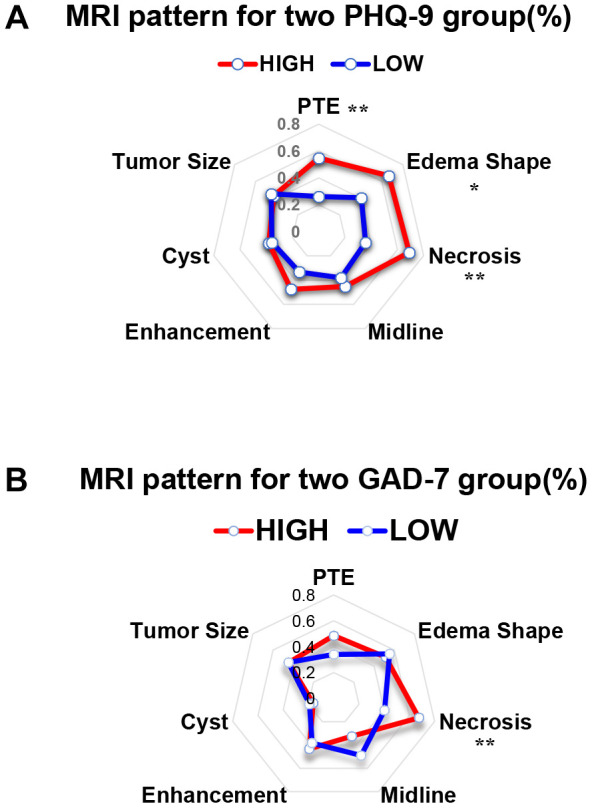
**The MRI results of patients with different PHQ-9 and GAD-7 scores presented different features.** (**A**) Radar chart showing the MRI features of the high and low PHQ-9 groups. Chi-squared analysis was conducted as the statistical method. **, *P* < 0.01; *, *P* < 0.05. (**B**) Radar chart showing the MRI features of the high and low GAD-7 groups. Chi-squared analysis was conducted as the statistical method. **, *P* < 0.01.

Our previous research demonstrated that different MRI patterns reflect the prognoses of GBM patients [19]; thus, we collected MRI images from patients in the present study (Supplementary Figure 3). As shown in Table 5, a significantly greater proportion of patients presented with large peritumor edemas, irregular edema shapes and necrotic tumors in the high PHQ-9 group than in the low PHQ-9 group; however, there was no significant difference in the proportion of patients whose tumor or edema crossed the midline of the brain, who exhibited contrast enhancement or who had a tumor larger than the median size. As for the GAD-7 scoring groups, we observed a significantly greater proportion of patients with necrotic tumors in the high GAD-7 group than in the low GAD-7 group. Radar charts were produced to more vividly depict the MRI characteristics of the different groups (Table 5, Figure 3A and 3B).

**Table 5:** MRI features of the high and low PHQ-9 and GAD-7 groups.

**Items**	**PTE**	**Edema shape**	**Necrosis**	**Midline**	**Enhancement**	**Cyst**	**Tumor size**
**PHQ-9**							
**High**	23(55%)	28(67%)	29(69%)	19(45%)	20(48%)	6(14%)	18(43%)
**Low**	11(26%)	17(40%)	15(36%)	16(38%)	14(33%)	7(17%)	19(45%)
***P* value**	0.008	0.016	0.002	0.507	0.182	0.763	0.826
**GAD-7**							
**High**	19(51%)	19(51%)	26(70%)	13(35%)	16(43%)	7(19%)	16(43%)
**Low**	16(34%)	26(55%)	19(40%)	23(49%)	18(38%)	9(19%)	21(44%)
***P* value**	0.100	0.717	0.006	0.204	0.647	0.979	0.895

The number and proportion of patients exhibiting each MRI feature is presented. PTE: peritumor edema ≥ 1 cm. Edema shape: irregularly shaped edema. Necrosis: tumor containing necrosis. Midline: tumor and/or edema that crossed the midline of the brain. Enhancement: contrast enhancement within the tumor. Cyst: a cyst within the tumor. Tumor size: tumor size greater than median. Pearson’s chi-squared test was conducted as the statistical method.

### Differences in cell biology between the high and low PHQ-9/GAD-7 groups

GBM cells with more aggressive proliferation and invasion abilities may be more likely to cause necrosis within the tumor itself [18, 20]. Our MRI images revealed that the proportion of patients with necrotic tumors was greater in the high PHQ-9 and GAD-7 groups than in the corresponding low groups. To further explore this phenomenon, we conducted cellular experiments using primary GBM cells from patients who obtained high or low scores on both the PHQ-9 and GAD-7 questionnaires.

**Figure 4:**
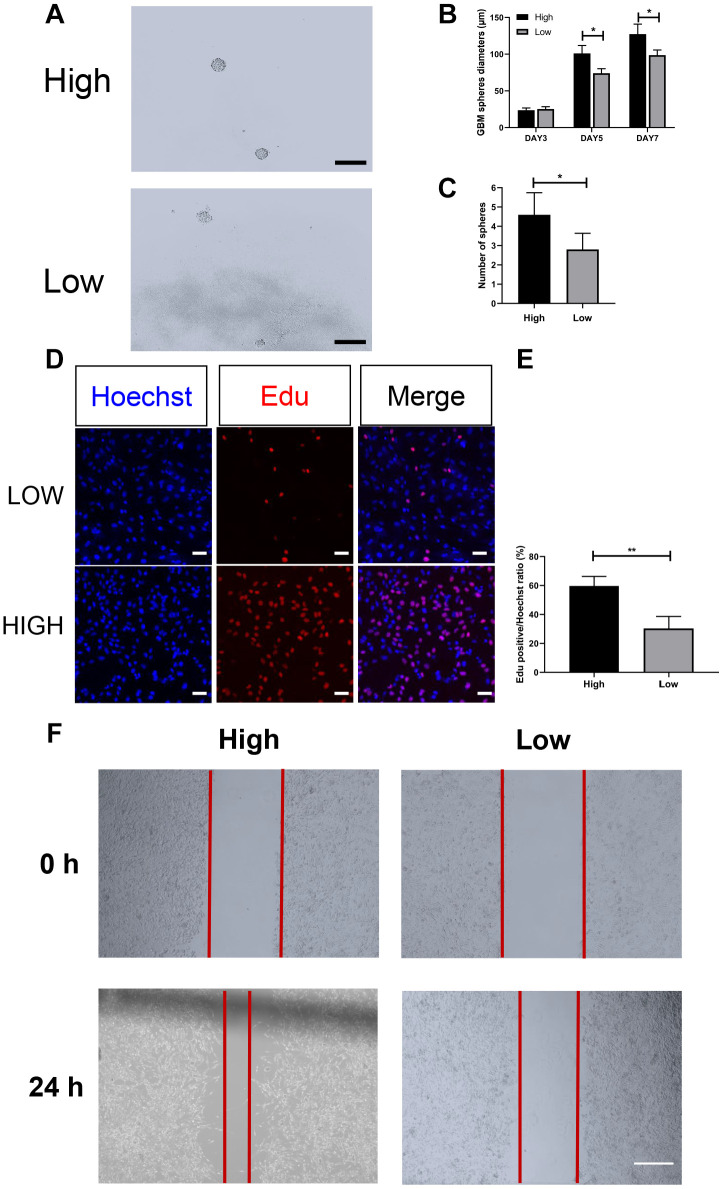
**Cellular experiments revealed differing proliferation and invasion abilities in the high- and low-score groups.** (**A**–**C**) Morphology of GBM spheres in the high and low PHQ-9/GAD-7 groups. The diameters of the GBM spheres in both groups increased gradually on days 3, 5 and 7, but those in the high group grew significantly faster (high group, n = 3; low group, n = 3). The number of spheres that developed from single cells was significantly greater in the high group than in the low group (*P* = 0.0216, n = 5). Data are presented as the mean ± standard error, and Student's t-test was chosen as the statistical method; *, *P* < 0.05. Scale bar: 200 μm. (**D**, **E**) The proliferation capacities of primary GBM cells from the different groups were evaluated with an EdU assay. Primary GBM cells from the low group (top) and high group (bottom) were stained with EdU (red, middle) and counterstained with Hoechst (blue, left). The ratio of EdU-positive cells to the total number of Hoechst-labeled cells is presented as the mean ± standard error, and Student's t-test was chosen as the statistical method; ***, *P* < 0.001, n = 3. Scale bar: 20 μm. (**F**) Wound healing assay of primary GBM cells from the high and low PHQ-9/GAD-7 groups. A six-well plate was scratched, and primary cells from the different groups were photographed at 0 and 24 h.

Sphere formation assays are commonly used to detect differences in cell proliferation. As shown in Figure 4A–4C, the sphere formation ability was significantly greater in the high PHQ-9/GAD-7 score group than in the low group. Although the mean diameter of the spheres did not differ significantly between the high and low groups on day 3 of cell culture, the growth rate of the diameter of the spheres within seven days was significantly faster in the high group than in the low group (high group: day 3, 23.67 ± 1.77 mm, day 5, 101.12 ± 6.25 mm, day 7, 127.33 ± 7.89 mm, n = 3; low group: day 3, 25.33 ± 1.77 mm, day 5, 74.00 ± 3.61 mm, day 7, 98.61 ± 4.06 mm, n = 3). After seven days of cell culture, we also calculated the number of spheres that developed from single cells in both groups. The number of spheres was significantly greater in the high group than in the low group (high group: 4.6 ± 0.51, n = 5; low group: 2.8 ± 0.37, n = 5). In addition, we observed a significantly greater proportion of 5-ethynyl-2-deoxyuridine (EdU)-positive cells in the high group than in the low group (Figure 4D and 4E). These results indicated that primary GBM cells from the high group had stronger proliferation abilities than those from the low group.

We also conducted a wound healing assay to compare the invasive abilities of primary GBM cells from the two groups. After 24 hours, we observed significantly better wound healing in the high group than in the low group (Figure 4F). These results indicated that GBM cells from the high group had greater invasive capacities than those from the low group.

### Expression patterns of mesenchymal and epithelial markers in both groups

**Figure 5:**
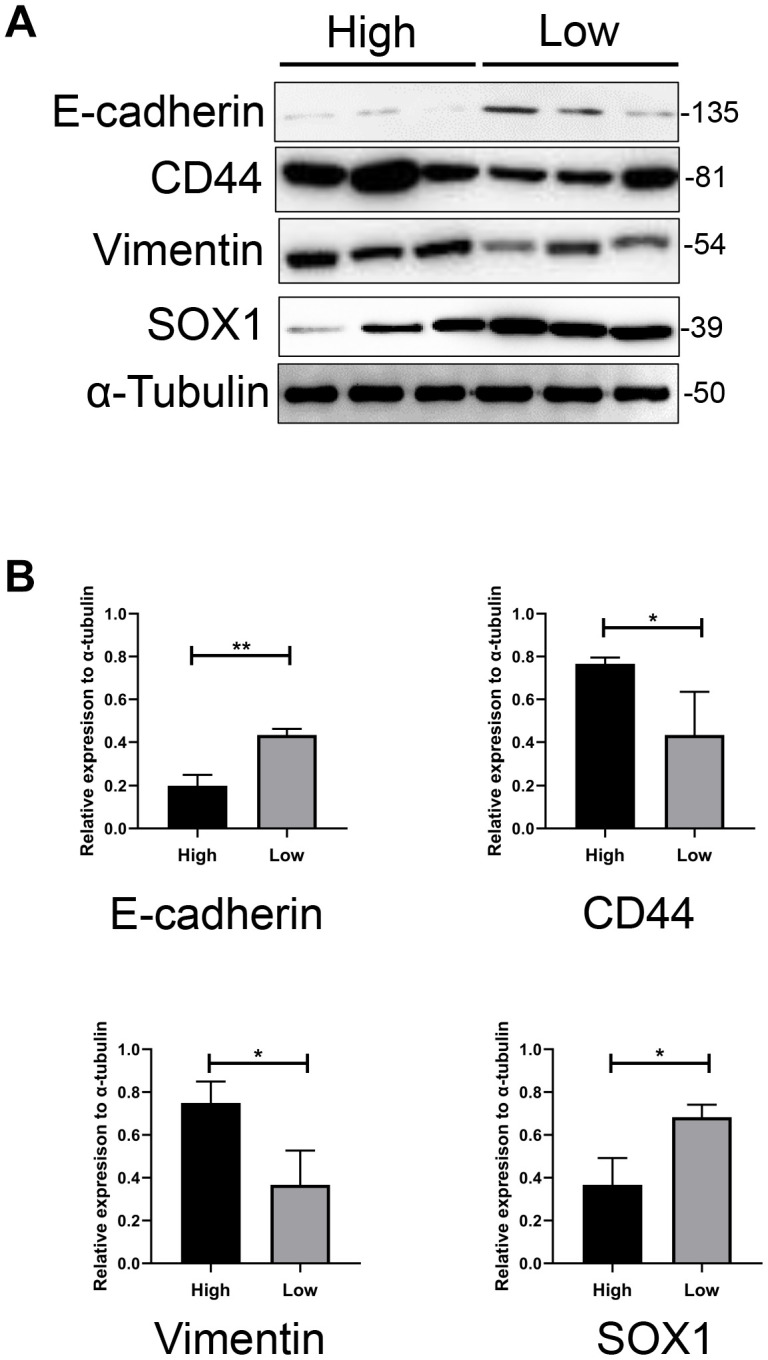
**The expression pattern of mesenchymal and epithelial markers in both groups.** (**A**) Western blotting assay showed significantly higher expression of CD44 and Vimentin, and lower expression of Sox1 and E-cadherin in GBM cells from higher scored group than lower scored group. α-tubulin was used as internal reference. (**B**) Bar charts showed the relative expression of these markers to α-tubulin and revealed the statistically different expression of these markers between two groups.

GBM has been divided into different subgroups, namely proneural, classical and mesenchymal subgroups, of which the mesenchymal subgroup displays the most aggressive and proliferative cell characteristics both *in vivo* and *in vitro*, as well as the worst patient prognosis [21]. Stimuli such as inflammatory responses, immune factors, etc. can induce the transformation of other GBM subgroups (especially the proneural subgroup) into the mesenchymal subgroup [22]. This process is called the proneural-mesenchymal transition, which is essentially the epithelial-mesenchymal transition (EMT) of GBM [23]. We examined the expression of mesenchymal (Vimentin and CD44) and epithelial markers (E-cadherin and SRY-Box Transcription Factor 1 [SOX1]) in cultured GBM cells from the high and low PHQ-9/GAD-7 score groups. As shown in Figure 5A and 5B, Vimentin and CD44 levels were significantly higher in the high-score group than in the low-score group, while E-cadherin and SOX1 levels were significantly lower in the high-score group than in the low-score group.

## DISCUSSION

Although psychosomatic diseases are a hot topic, the relationship between psychological disorders and physical diseases (especially cancers) has remained unclear. Cancer diagnosis and treatment critically impact the psychological well-being of patients. The development of GBM, the most common type of malignant tumor in the human brain, could be one of the most serious incidents in a person’s life, and could also potentially cause psychological decay. Using a questionnaire survey based on the PHQ-9 scale, Alasdair italic>et al. found that glioma patients, especially grade IV glioma (namely GBM) patients, were prone to developing depressive disorder [12]. However, the development of a psychological disorder is a complex process that often involves the sum of multiple emotional changes, rather than a single emotional change. It is believed that depressive and anxiety disorders always develop simultaneously in patients confronting severe physical problems such as traumatic diseases and cancers. Thus, researchers typically use the GAD-7 scale together with the PHQ-9 scale to assess the mental situation of patients with traumatic brain injuries [10], multiple sclerosis [24], etc. For this reason, we used both of these questionnaires in our research.

We discovered that patients who had learned of their GBM diagnoses had higher PHQ-9 and GAD-7 scores than healthy controls, and some patients even exhibited moderate depression and anxiety. GBM patients could understandably have violent emotional swings that even develop into mental changes after receiving such serious negative news. Thus, what really interested us was the diversity of psychological states among GBM patients, and the possibility that these differences would significantly correlate with the patients’ prognoses. We found that patients with higher PHQ-9 and GAD-7 scores had poorer prognoses than those with lower scores.

In addition to the rationale mentioned above, we selected the PHQ-9 and GAD-7 scales because we wanted to accommodate patients from various age and educational backgrounds (our GBM patients ranged from around 40 to 60 years old). Although these two scales are widely accepted and used in psychological and clinical investigations, they may cause some bias because they contain relatively few items. Despite this limitation, we still obtained convincing results, and future experiments should allow us to draw more comprehensive conclusions about the relationship between depressive/anxiety disorders and GBM prognosis.

We also noticed that female GBM patients had higher PHQ-9 scores than male patients, although GAD-7 scores did not differ significantly between the genders. Moreover, male patients with high PHQ-9 and GAD-7 scores had significantly shorter overall survival times than those with low scores, while female patients only exhibited survival differences based on their GAD-7 scores. Many studies have indicated that women tend to be more sensitive than men to the same mental assaults, and are more prone to developing depressive disorder [25, 26]. There are likely multiple reasons for these phenomena. The large amplitudes of emotional fluctuations in female patients are not always directly related to the severity of their mental conditions. On the other hand, the psychological symptoms of male patients are often concealed by various complex factors. Thus, when male patients finally show symptoms of depression or anxiety, they have already reached a relatively heavy level of mental illness. It is unclear whether endocrine differences between the genders contribute to these trends; however, endocrine factors have been reported to impact the development of depression, anxiety [27, 28] and tumors [29, 30]. Nevertheless, many factors could have influenced the relationship between PHQ-9/GAD-7 results and GBM prognosis, so these results should be further confirmed in future studies.

Although it seems plausible that the sudden negative news of a GBM diagnosis could induce the development of psychological disorders, we could not determine whether the news of the tumor diagnosis or the cellular/molecular changes in the tumor itself led to the patients’ depression and anxiety symptoms. Patients with higher scores on the two scales had poorer prognoses after tumor resection, suggesting that the biological mechanisms of depression and anxiety also participated in the development and outcomes of GBM. To explore the reasons for the prognostic differences between patients with high and low PHQ-9 and GAD-7 scores, we performed MRI and cellular studies.

We observed that patients’ MRI results partly reflected the pathology pattern of GBM. Patients with higher PHQ-9 and GAD-7 scores were more likely to exhibit necrosis within their tumors than patients with lower scores. GBM neovascularization often occurs at the interface with normal tissues, so rapidly proliferating tumor cells may induce necrosis due to the distribution of new blood vessels. When this occurs, unique cystic necrosis and circular enhancement can be observed upon MRI [3, 18, 31], and these imaging characteristics often indicate a poor prognosis [1, 3]. Thus, our MRI findings may explain why patients with higher depression and anxiety scores had poorer prognoses. However, imaging evidence alone would not be sufficient to explain this phenomenon, so we also conducted cellular experiments.

When we cultured primary GBM cells from patients with high or low scores on both the PHQ-9 and GAD-7 scales, we discovered that cells from the high-score group exhibited greater proliferation and invasion abilities than those from the low-score group. These findings may partly explain the association of higher PHQ-9 and GAD-7 scores with a poorer prognosis. Elevated levels of pro-inflammatory cytokines (e.g., interleukin-6, tumor necrosis factor α, interferon α, interferon γ and transforming growth factor β) and neurotransmitters (e.g., dopamine, norepinephrine and serotonin) may be crucial contributors to the development of both depressive/anxiety disorders and glioma [30, 32–35]. In GBM, for example, histamine induces cell membrane hyperpolarization, serotonin promotes cellular growth, norepinephrine inhibits glucose uptake, dopamine is associated with cell proliferation, and so forth [30, 34]. Cytokines such as tumor necrosis factor α and transforming growth factor β, which are involved in depressive and anxiety disorders, may also initiate the EMT in GBM [36]. The EMT, in turn, could increase the aggressiveness, invasiveness, proliferation and migration of GBM cells, leading to different MRI features and poorer prognoses in GBM patients [21, 37–39].

To further explore this possibility, we measured the expression of mesenchymal (Vimentin and CD44) and epithelial markers (E-cadherin and SOX1) in GBM cells from the high and low PHQ-9/GAD-7 score groups. Mesenchymal marker levels were greater in the high-score group than in the low-score group, which may explain the higher cellular growth and migration rates, the greater proportion of necrotic tumors and the poorer outcomes in the high-score group. We suppose that microenvironmental alterations in the brain may have both increased patients’ sensitivity to depressive/anxiety disorders and induced the EMT of GBM in the high-score group. However, additional evidence is needed to support this speculation. Therefore, in future research, we will collect additional tumor samples from GBM patients for more comprehensive experiments. Genomic or even single-cell sequencing of samples may provide further insight.

In conclusion, our research demonstrated that GBM patients had higher PHQ-9 and GAD-7 scores than healthy controls. Among GBM patients, those with higher scores on these scales had poorer prognoses than those with lower scores. MRI results from GBM patients revealed that tumor necrosis was more common in the high-score groups, suggesting that GBM cells from the high-score groups proliferated at a faster rate than those from the low-score groups. Cellular experiments confirmed that GBM cells from patients with higher PHQ-9 and GAD-7 scores had greater proliferation and invasion abilities than those from patients with lower scores. The higher Vimentin and CD44 levels in GBM cells from high-score patients, indicating the presence of more cells in the mesenchymal subgroup, may explain the greater aggressiveness of GBM cells from these patients.

## MATERIALS AND METHODS

### Subjects

We enrolled a total of 84 patients (40 men and 44 women) ranging from 32 to 68 (44.11 ± 1.09) years old with pathological diagnoses of GBM in our project. All the enrolled GBM patients had Karnofsky Performance Status scores greater than 70. For the controls, we randomly selected 45 healthy adults from different backgrounds (26 men and 19 women) ranging from 33 to 63 (46.02 ± 1.12) years old. This study was approved by the local Ethics Committee and conformed to the principles outlined in the Declaration of Helsinki. All patients provided written informed consent.

### Instruments

In order to accommodate the different educational backgrounds of patients of different ages, we selected the PHQ-9 and GAD-7 scales to assess the presence of major depressive disorder and anxiety disorder. These scales are widely used as reliable measures of depression and anxiety [10, 40]. All patients were asked to complete the two questionnaires after being diagnosed with glioma (even though the diagnosis of GBM had not yet been pathologically verified). For the control group, a community survey was conducted. The detailed items of the PHQ-9 and GAD-7 surveys are presented in Tables 1 and 2.

### MRI feature definition

The definitions of MRI features in GBM patients were detailed in our previous article [19]. In short, for all patients, the unidimensional maximum diameter in centimeters was used to measure the tumor size on T1-W images. The median tumor size was 4.8 cm (ranging from 2.2 to 9.9 cm). In accordance with the method of Hartmann [41] peritumor edema was morphologically classified based of T2-W images, and the severity was determined based on whether the edema was ≥ 1 cm or < 1 cm. Necrosis was assessed on axial contrast-enhanced T1-W images, and was established when a region had a high signal on T2-W images, a low signal on T1-W images and an irregular enhancing border on contrast-enhanced images. A cyst was defined as a rounded region with a low T1-W signal, a very high T2-W signal matching a cerebrospinal fluid signal, and a thin, smooth, regular and slightly enhancing or non-enhancing wall. Contrast enhancement of a tumor was classified as not obvious (less than the signal of fat) or obvious (similar to that of fat). The specific classification of imaging features is described in Supplementary Table 2. Using these classification methods, two experienced radiologists without knowledge of our research independently analyzed the imaging data of all patients.

### Culture of primary GBM cells

Briefly, jelly-like tumor tissue was obtained during surgery and placed in a sterilized 50-mL centrifuge tube containing ice-cold phosphate-buffered saline. Then, the tumor tissue was carefully transported from the operating room to the laboratory in an icebox. The supernatant was discarded, and the tissue sample was placed in a sterile dish and cut into 1-mm^3^ pieces with sterilize scissors and tweezers. The tissue pieces were transferred to a 15-mL centrifuge tube, and phosphate-buffered saline containing 1% penicillin-streptomycin was added. The sample was mixed and shaken up and down three times to remove any remaining red blood cells as thoroughly as possible. Once the upper layer of liquid became clear, the supernatant was carefully removed and about 3 mL of trypsin was added for every 2 cm^3^ of tissue. The sample was incubated at 37 °C for 10 min and shaken every 2 min to ensure that the tissue was fully digested. After the digestion, the tube was allowed to stand for 2 min before the supernatant was transferred to an Eppendorf tube and centrifuged for 5 min at 1000 rpm.

After the centrifugation, the pelleted cells were resuspended in Dulbecco’s Modified Eagle’s Medium containing 10% fetal bovine serum, and were transferred to a culture dish. The cells were incubated at 37 °C with 5% CO_2_, and the medium was changed every two days. The primary GBM cells appeared as irregular spindle-like cells under a microscope. Primary GBM cells were cultured in stem cell culture medium – either Dulbecco’s Modified Eagle’s Medium or neurobasal medium (Life Technologies Corporation) supplemented with 20 ng/mL fibroblast growth factor 2 (PeproTech), 20 ng/mL epidermal growth factor (PeproTech), B27 and N2 supplemental factors (Gibco) and antibiotics (penicillin and streptomycin). Isolated GBM cells grew into spheres after one to two months of continuous culturing. GBM stem cells were tested for their capability to self-renew using the sphere-formation assay described below.

### EdU assay

The EdU assay was conducted according to the manufacturer’s instructions. Briefly, primary GBM cells from the different groups were plated and cultured in a 12-well culture plate with a cover glass. The cells were then treated with 50 mM EdU for 2 h, followed by Azide-555 staining and DNA staining. Images were acquired and analyzed with FLUOVIEW FV 10i (Olympus).

### Sphere formation assay

GBM spheres were harvested and dissociated with 0.25% trypsin. After centrifugation, the suspensions of expanded GBM cells were seeded in a 96-well plate (three to four cells per well). On days 3, 5 and 7 of incubation in GBM stem cell proliferation medium, the long diameter and shape of each sphere was measured. A digital still camera (Axio Observer A1) was used to convert the fixed area (10 mm^2^) at the center of each well into a digital image, and Image-Pro Plus 6.0 (Media Cybernetics) was used to count the number of GBM cell spheres.

### Western blotting

Western blotting was performed on primary GBM cells. Briefly, 50 mg of total protein from each group was separated on a 10% sodium dodecyl sulfate polyacrylamide gel and transferred to a 0.22-mm polyvinylidene difluoride membrane (Millipore). The membrane was blocked with 5% skim milk at room temperature for 2 h and then incubated with specific primary antibodies at 4 °C overnight. The membrane was then incubated with the appropriate horseradish peroxidase-conjugated secondary antibody (diluted 1:5000; Boster) at 37 °C for 1 h. Protein bands on the membrane were visualized with an enhanced chemiluminescence kit (Millipore) using a FluorChem FC system (Alpha Innotech Corporation).

### Statistical tests

All variables are presented as the mean ± standard error. Since the variables were normally distributed, we used Student’s t-test or one-way analysis of variance for statistical analysis. GraphPad Prism software was used (Version 5.01; GraphPad Software, Inc). *P* < 0.05 was considered statistically significant.

## Supplementary Material

Supplementary Figures

Supplementary Tables

## References

[1] Louis DN, Perry A, Reifenberger G, von Deimling A, Figarella-Branger D, Cavenee WK, Ohgaki H, Wiestler OD, Kleihues P, Ellison DW. The 2016 world health organization classification of tumors of the central nervous system: a summary. Acta Neuropathol. 2016; 131:803–20. 10.1007/s00401-016-1545-1 27157931

[2] de Robles P, Fiest KM, Frolkis AD, Pringsheim T, Atta C, St Germaine-Smith C, Day L, Lam D, Jette N. The worldwide incidence and prevalence of primary brain tumors: a systematic review and meta-analysis. Neuro Oncol. 2015; 17:776–83. 10.1093/neuonc/nou283 25313193 PMC4483114

[3] Hanif F, Muzaffar K, Perveen K, Malhi SM, Simjee SU. Glioblastoma multiforme: a review of its epidemiology and pathogenesis through clinical presentation and treatment. Asian Pac J Cancer Prev. 2017; 18:3–9. 10.22034/APJCP.2017.18.1.3 28239999 PMC5563115

[4] Chow D, Chang P, Weinberg BD, Bota DA, Grinband J, Filippi CG. Imaging genetic heterogeneity in glioblastoma and other glial tumors: review of current methods and future directions. AJR Am J Roentgenol. 2018; 210:30–38. 10.2214/AJR.17.18754 28981352

[5] Santini ZI, Jose PE, York Cornwell E, Koyanagi A, Nielsen L, Hinrichsen C, Meilstrup C, Madsen KR, Koushede V. Social disconnectedness, perceived isolation, and symptoms of depression and anxiety among older Americans (NSHAP): a longitudinal mediation analysis. Lancet Public Health. 2020; 5:e62–70. 10.1016/S2468-2667(19)30230-0 31910981

[6] Forero DA, Guio-Vega GP, González-Giraldo Y. A comprehensive regional analysis of genome-wide expression profiles for major depressive disorder. J Affect Disord. 2017; 218:86–92. 10.1016/j.jad.2017.04.061 28460316

[7] Tang W, Lu Y, Xu J. Post-traumatic stress disorder, anxiety and depression symptoms among adolescent earthquake victims: comorbidity and associated sleep-disturbing factors. Soc Psychiatry Psychiatr Epidemiol. 2018; 53:1241–51. 10.1007/s00127-018-1576-0 30109368

[8] Gialluisi A, Bonaccio M, Di Castelnuovo A, Costanzo S, De Curtis A, Sarchiapone M, Cerletti C, Donati MB, de Gaetano G, Iacoviello L, Moli-Sani Study Investigators. Lifestyle and biological factors influence the relationship between mental health and low-grade inflammation. Brain Behav Immun. 2020; 85:4–13. 10.1016/j.bbi.2019.04.041 31055172

[9] Treudler R, Zeynalova S, Riedel-Heller SG, Zuelke AE, Roehr S, Hinz A, Glaesmer H, Kage P, Loeffler M, Simon JC. Depression, anxiety and quality of life in subjects with atopic eczema in a population-based cross-sectional study in Germany. J Eur Acad Dermatol Venereol. 2020; 34:810–16. 10.1111/jdv.16148 31838777

[10] Teymoori A, Real R, Gorbunova A, Haghish EF, Andelic N, Wilson L, Asendorf T, Menon D, von Steinbüchel N. Measurement invariance of assessments of depression (PHQ-9) and anxiety (GAD-7) across sex, strata and linguistic backgrounds in a european-wide sample of patients after traumatic brain injury. J Affect Disord. 2020; 262:278–85. 10.1016/j.jad.2019.10.035 31732280

[11] Mulugeta A, Zhou A, King C, Hyppönen E. Association between major depressive disorder and multiple disease outcomes: a phenome-wide mendelian randomisation study in the UK Biobank. Mol Psychiatry. 2020; 25:1469–76. 10.1038/s41380-019-0486-1 31427754

[12] Rooney AG, McNamara S, Mackinnon M, Fraser M, Rampling R, Carson A, Grant R. Screening for major depressive disorder in adults with cerebral glioma: an initial validation of 3 self-report instruments. Neuro Oncol. 2013; 15:122–29. 10.1093/neuonc/nos282 23229997 PMC3534425

[13] Young K, Singh G. Biological mechanisms of cancer-induced depression. Front Psychiatry. 2018; 9:299. 10.3389/fpsyt.2018.00299 30042700 PMC6048357

[14] Morawa E, Erim Y. Depressive complaints and utilization of mental health services: comparison of adult cancer survivors of different ethnic origin. J Psychosom Res. 2020; 130:109915. 10.1016/j.jpsychores.2019.109915 31918358

[15] Pitman A, Suleman S, Hyde N, Hodgkiss A. Depression and anxiety in patients with cancer. BMJ. 2018; 361:k1415. 10.1136/bmj.k1415 29695476

[16] Sherman KA, Przezdziecki A, Alcorso J, Kilby CJ, Elder E, Boyages J, Koelmeyer L, Mackie H. Reducing body image-related distress in women with breast cancer using a structured online writing exercise: results from the my changed body randomized controlled trial. J Clin Oncol. 2018; 36:1930–40. 10.1200/JCO.2017.76.3318 29688834

[17] Satin JR, Linden W, Phillips MJ. Depression as a predictor of disease progression and mortality in cancer patients: a meta-analysis. Cancer. 2009; 115:5349–61. 10.1002/cncr.24561 19753617

[18] Aldape K, Zadeh G, Mansouri S, Reifenberger G, von Deimling A. Glioblastoma: pathology, molecular mechanisms and markers. Acta Neuropathol. 2015; 129:829–48. 10.1007/s00401-015-1432-1 25943888

[19] Wu CX, Lin GS, Lin ZX, Zhang JD, Liu SY, Zhou CF. Peritumoral edema shown by MRI predicts poor clinical outcome in glioblastoma. World J Surg Oncol. 2015; 13:97. 10.1186/s12957-015-0496-7 25886608 PMC4358863

[20] Jackson CM, Choi J, Lim M. Mechanisms of immunotherapy resistance: lessons from glioblastoma. Nat Immunol. 2019; 20:1100–09. 10.1038/s41590-019-0433-y 31358997

[21] Lah TT, Novak M, Breznik B. Brain Malignancies: glioblastoma and brain metastases. Semin Cancer Biol. 2020; 60:262–73. 10.1016/j.semcancer.2019.10.010 31654711

[22] Ozawa T, Riester M, Cheng YK, Huse JT, Squatrito M, Helmy K, Charles N, Michor F, Holland EC. Most human non-GCIMP glioblastoma subtypes evolve from a common proneural-like precursor glioma. Cancer Cell. 2014; 26:288–300. 10.1016/j.ccr.2014.06.005 25117714 PMC4143139

[23] Lamouille S, Xu J, Derynck R. Molecular mechanisms of epithelial-mesenchymal transition. Nat Rev Mol Cell Biol. 2014; 15:178–96. 10.1038/nrm3758 24556840 PMC4240281

[24] Hua LH, Fan TH, Conway D, Thompson N, Kinzy TG. Discontinuation of disease-modifying therapy in patients with multiple sclerosis over age 60. Mult Scler. 2019; 25:699–708. 10.1177/1352458518765656 29557704

[25] Gao W, Ping S, Liu X. Gender differences in depression, anxiety, and stress among college students: a longitudinal study from China. J Affect Disord. 2020; 263:292–300. 10.1016/j.jad.2019.11.121 31818792

[26] Salk RH, Hyde JS, Abramson LY. Gender differences in depression in representative national samples: meta-analyses of diagnoses and symptoms. Psychol Bull. 2017; 143:783–822. 10.1037/bul0000102 28447828 PMC5532074

[27] Nader N, Chrousos GP, Kino T. Interactions of the circadian CLOCK system and the HPA axis. Trends Endocrinol Metab. 2010; 21:277–86. 10.1016/j.tem.2009.12.011 20106676 PMC2862789

[28] Sundström Poromaa I, Comasco E, Georgakis MK, Skalkidou A. Sex differences in depression during pregnancy and the postpartum period. J Neurosci Res. 2017; 95:719–30. 10.1002/jnr.23859 27870443 PMC5129485

[29] Fu X, Pereira R, De Angelis C, Veeraraghavan J, Nanda S, Qin L, Cataldo ML, Sethunath V, Mehravaran S, Gutierrez C, Chamness GC, Feng Q, O’Malley BW, et al. FOXA1 upregulation promotes enhancer and transcriptional reprogramming in endocrine-resistant breast cancer. Proc Natl Acad Sci USA. 2019; 116:26823–34. 10.1073/pnas.1911584116 31826955 PMC6936436

[30] Hujber Z, Horváth G, Petővári G, Krencz I, Dankó T, Mészáros K, Rajnai H, Szoboszlai N, Leenders WP, Jeney A, Tretter L, Sebestyén A. GABA, glutamine, glutamate oxidation and succinic semialdehyde dehydrogenase expression in human gliomas. J Exp Clin Cancer Res. 2018; 37:271. 10.1186/s13046-018-0946-5 30404651 PMC6223071

[31] Gokden M. If it is not a glioblastoma, then what is it? a differential diagnostic review. Adv Anat Pathol. 2017; 24:379–91. 10.1097/PAP.0000000000000170 28885262

[32] Amoroso M, Böttcher A, Lowry CA, Langgartner D, Reber SO. Subcutaneous Mycobacterium vaccae promotes resilience in a mouse model of chronic psychosocial stress when administered prior to or during psychosocial stress. Brain Behav Immun. 2020; 87:309–17. 10.1016/j.bbi.2019.12.018 31887415

[33] Franklin TC, Xu C, Duman RS. Depression and sterile inflammation: essential role of danger associated molecular patterns. Brain Behav Immun. 2018; 72:2–13. 10.1016/j.bbi.2017.10.025 29102801

[34] Johanns TM, Miller CA, Liu CJ, Perrin RJ, Bender D, Kobayashi DK, Campian JL, Chicoine MR, Dacey RG, Huang J, Fritsch EF, Gillanders WE, Artyomov MN, et al. Detection of neoantigen-specific T cells following a personalized vaccine in a patient with glioblastoma. Oncoimmunology. 2019; 8:e1561106. 10.1080/2162402X.2018.1561106 30906654 PMC6422384

[35] Lasselin J. Is inflammation-associated depression atypical depression? Brain Behav Immun. 2020; 87:193–94. 10.1016/j.bbi.2020.01.008 31926289

[36] Colella B, Faienza F, Di Bartolomeo S. EMT regulation by autophagy: a new perspective in glioblastoma biology. Cancers (Basel). 2019; 11:312. 10.3390/cancers11030312 30845654 PMC6468412

[37] Segerman A, Niklasson M, Haglund C, Bergström T, Jarvius M, Xie Y, Westermark A, Sönmez D, Hermansson A, Kastemar M, Naimaie-Ali Z, Nyberg F, Berglund M, et al. Clonal variation in drug and radiation response among glioma-initiating cells is linked to proneural-mesenchymal transition. Cell Rep. 2016; 17:2994–3009. 10.1016/j.celrep.2016.11.056 27974212

[38] Williams ED, Gao D, Redfern A, Thompson EW. Controversies around epithelial-mesenchymal plasticity in cancer metastasis. Nat Rev Cancer. 2019; 19:716–32. 10.1038/s41568-019-0213-x 31666716 PMC7055151

[39] Naeini KM, Pope WB, Cloughesy TF, Harris RJ, Lai A, Eskin A, Chowdhury R, Phillips HS, Nghiemphu PL, Behbahanian Y, Ellingson BM. Identifying the mesenchymal molecular subtype of glioblastoma using quantitative volumetric analysis of anatomic magnetic resonance images. Neuro Oncol. 2013; 15:626–34. 10.1093/neuonc/not008 23444259 PMC3635524

[40] Kroenke K, Spitzer RL, Williams JB. The PHQ-9: validity of a brief depression severity measure. J Gen Intern Med. 2001; 16:606–13. 10.1046/j.1525-1497.2001.016009606.x 11556941 PMC1495268

[41] Hartmann M, Jansen O, Egelhof T, Forsting M, Albert FK, Sartor K. [Effect of brain edema on the recurrence pattern of Malignant gliomas]. {Article in German]. Radiologe. 1998; 38:948–53. 10.1007/s001170050447 9861656

