# Supplementary material for: Depressive and anxiety disorders worsen the prognosis of glioblastoma
Source: Aging (Albany NY). 2020 Oct 28;12(20):20095–110. doi: 10.18632/aging.103593 (PMC7655183; doi:10.18632/aging.103593)
Supplement: Supplementary Figures [file aging-12-103593-s001..pdf]

## SUPPLEMENTARY FIGURES

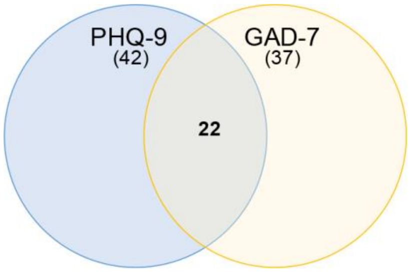


**Supplementary Figure 1. Patients gained higher scores of both the two scales.**

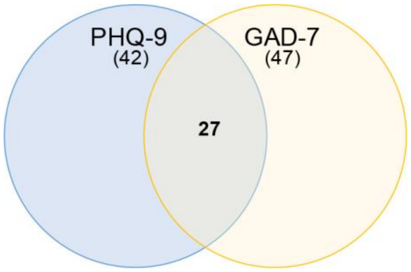


**Supplementary Figure 2. Patients gained lower scores of both the two scales.**

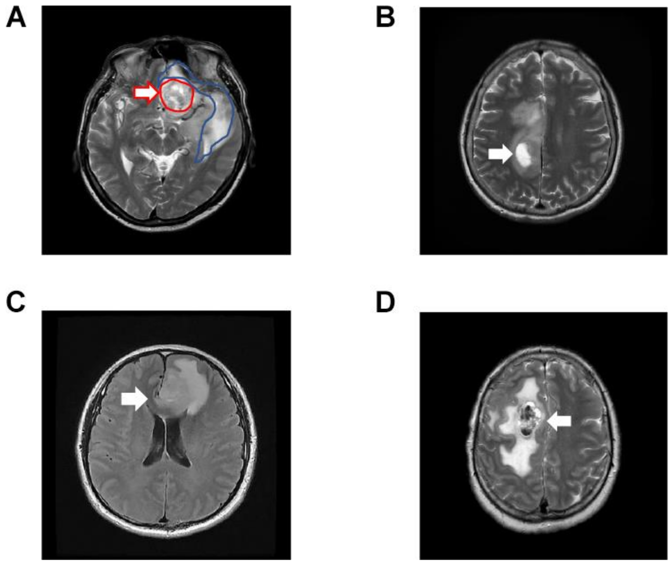


**Supplementary Figure 3. Magnetic Resonance Imaging (MRI) figure shows different patterns of GBM patient.** (**A**) Tumor body (red arrow) and peritumor edema (blue arrow); (**B**) Cyst with a tumor (white arrow); (**C**) Tumor and edema cross midline (white arrow); (**D**) Necrosis within a tumor (white arrow).
