# Supplementary material for: Depressive and anxiety disorders worsen the prognosis of glioblastoma
Source: Aging (Albany NY). 2020 Oct 28;12(20):20095–110. doi: 10.18632/aging.103593 (PMC7655183; doi:10.18632/aging.103593)
Supplement: Supplementary Tables [file aging-12-103593-s002..pdf]

SUPPLEMENTARY TABLES

Supplementary Table 1. Basic information of enrolled patients and control group.

	GBM	CONTROL	P value
**Total**	84	45	-
**Male (%)**	40	24	0.582
**Female (%)**	44	21	0.582
**Age**	45.04±0.88(years)	46.02±1.71(years)	0.580

There was no significant difference between the two groups in terms of gender (Pearson Chi-square test, $P = 0.582$) and age (Student t test, $P = 0.580$). GBM: Glioblastoma Multiforme.

Supplementary Table 2. Definition of MRI patterns.

Imaging features	Classification criterion
**Edema extent**	
**Minor**	Peritumoral edema extending <1 cm from tumor margin
**Major**	Peritumoral edema extending ≥1 cm from tumor margin
**Edema shape**	
**Regular**	The shape of edema is similar to round and is not radial
**Irregular**	The shape of edema tends to irregular, such as finger-like or radial shape
**Necrosis**	
**No**	No necrosis within tumor
**Yes**	A region had high signal on T2-W images, but low signal on T1-W images, and had an irregular enhancing border on contrast-enhanced images
**Cyst**	
**No**	No cyst in tumor
**Yes**	A rounded region which was very low T1-W signal and very high T2-W signal matching cerebrospinal fluid signal, and had a thin, smooth, regular, and slightly enhancing or non-enhancing wall
**Enhancement**	
**No obvious**	Enhancement signal is less than the signal of fat
**Obvious**	Enhancement signal is similar to that of fat
**Tumor crosses midline**	
**No**	Tumor is limited to unilateral cerebral hemisphere
**Yes**	Tumor crosses the brain midline and extends into the other side of cerebral hemisphere
**Edema crosses midline**	
**No**	Peritumoral edema extent is limited to unilateral cerebral hemisphere
**Yes**	Peritumoral edema extent crosses the brain midline and is not confined to unilateral cerebral hemisphere
**Size (cm)**	
**< median**	The maximum diameter of tumor is less than 4.8 cm
**≥median**	The maximum diameter of tumor is equal to or more than 4.8 cm
